# Product labeling accuracy and contamination analysis of commercially available cannabidiol product samples

**DOI:** 10.3389/fphar.2024.1335441

**Published:** 2024-03-18

**Authors:** Barry E. Gidal, Ryan Vandrey, Chela Wallin, Sean Callan, Alan Sutton, Timothy B. Saurer, Jennifer L. Triemstra

**Affiliations:** ^1^ University of Wisconsin School of Pharmacy, Madison, WI, United States; ^2^ Johns Hopkins University School of Medicine, Baltimore, MD, United States; ^3^ Ellipse Analytics, Denver, CO, United States; ^4^ Jazz Pharmaceuticals, Carlsbad, CA, United States

**Keywords:** cannabidiol, product label accuracy, contaminants, mislabeling, quality control

## Abstract

**Background and objective:** Commercially available cannabidiol (CBD) products are increasingly being used for medicinal purposes, including for the treatment of various neurological conditions, but there are growing concerns around adherence to quality control measures that protect consumers. This study was conducted to assess the purity and label accuracy of commercially available CBD products.

**Methods:** Commercially available CBD products were chosen from the open stream of commerce in the United States based on formulations as a tincture, gummy, vape, or topical product. Cannabinoid concentrations were analyzed to verify label accuracy including “full spectrum,” “broad spectrum,” and “CBD isolate” claims on the product label. Analysis for the presence of contaminants included evaluation for heavy metals, pesticides, and residual solvents. Labeled and actual total amounts of CBD and levels of impurities such as heavy metals, residual solvents, and pesticides were measured.

**Results:** A total of 202 CBD products (100 tinctures, 48 gummies, 34 vape products, and 20 topicals) were chosen to represent a broad sample in the United States. Of the products tested (full spectrum, n = 84; broad spectrum, n = 28; CBD isolate, n = 37), 26% did not meet the definition for product type claimed on the packaging. The majority of products (74%) deviated from their label claim of CBD potency by at least 10%. Heavy metals were detected 52 times across 44 of the 202 products tested, with lead being the most prevalent heavy metal. Residual solvents were detected 446 times across 181 of 202 products, with the highest concentrations reported for hexane, m/p-xylene, methanol, and o-xylene. Of 232 pesticides tested, 26 were found 55 times across 30 products. A total of 3% of heavy metals, 1% of residual solvents, and 1% of pesticides violated >1 regulatory threshold.

**Discussion:** This study demonstrated that the majority of commercially available CBD products tested within the current study are inaccurately labeled. Heavy metals, residual solvents, and pesticides were found in several products, some of which violated regulatory thresholds. Thus, uniform compliance with CBD quality control measures is lacking and raises consumer protection concerns. Improved regulatory oversight of this industry is recommended.

## Introduction

Phytocannabinoids derived from the *Cannabis sativa* L. plant are of considerable medical interest (Pertwee, 2014; Morales, et al., 2017). The two most well-characterized phytocannabinoids are delta-9-tetrahydrocannabinol (Δ9-THC or THC) and cannabidiol (CBD) (Ligresti, et al., 2016). Unlike THC, CBD is a non-intoxicating phytocannabinoid, and has shown affinity for various receptors (e.g., G protein-coupled receptor 55, equilibrative nucleoside transporter 1, transient receptor potential vanilloid 1, peroxisome proliferator-activated receptor gamma, and allosteric binding to cannabinoid receptors 1 & 2, not the orthostatic binding site) (Bisogno, et al., 2001; Carrier, et al., 2006; Peng, et al., 2022). Many health claims have been made for cannabinoids (Ligresti, et al., 2016). United States Food and Drug Administration-approved pharmaceutical formulations include one plant-derived CBD drug product and three synthetic THC drug products (EPIDIOLEX^®^ [prescribing information], 2023; MARINOL^®^ [prescribing information], 2017; SYNDROS^®^ [prescribing information], 2020; CESAMET^®^[NDA], 2006). Highly purified plant-derived CBD oral solution is approved in the United States and European Union for the treatment of seizures associated with several forms of rare epilepsy (EPIDIOLEX^®^ [prescribing information], 2023; EPIDYOLEX^®^ 100 mg/mL oral solution, 2019). With these proven, as well as perceived, health benefits, people are becoming increasingly interested in using cannabinoids, particularly CBD, for medicinal purposes.

The Agricultural Improvement Act of 2018 removed hemp with <0.3% THC from the list of controlled substances in the US, giving rise to a large hemp industry (Hudak J., 2018). Since then, the market for hemp products with high CBD content has grown considerably and is predicted to reach $16 billion by 2026 (Krawiec S., 2021), with over 1,500 active brands competing for market share (Nichols K., 2020). Commercialized hemp-sourced CBD products are available in a diverse array of formulations, including tinctures, gummies, capsules, food products, beverages, vape products, and topical products.

The hemp CBD product industry has faced significant challenges with quality assurance, including discrepancy in product labeling and presence of potentially toxic contaminants (Bonn-Miller, et al., 2017; Hazekamp, 2018; Poklis, et al., 2019; Gurley, et al., 2020; Wheeler, et al., 2020; Dunn, et al., 2021; Gardener, et al., 2022). Lead exposure can lead to substantial neurotoxic effects in children and adults (Sanders, et al., 2009; World Health Organization, 2022), and there is no known safe blood lead concentration (World Health Organization, 2022). Chronic cadmium exposure is associated with kidney (Prozialeck and Edwards, 2012), bone (Kazantzis, 2004), and lung diseases (Lampe, et al., 2008), while arsenic exposure can have deleterious effects on development and various organ systems, and has also been associated with multiple forms of cancer (Naujokas, et al., 2013). Residual solvents can have numerous harmful impacts on the human body, including neurotoxic effects with heptane and hexane (Kutlu, et al., 2009; Clough, 2014). Xylene has been shown to have ill effects on various systems of the body (Rajan and Malathi, 2014), and long-term exposure to methanol has a broad range of adverse effects on the eyes and the skin (The National Institute for Occupational Safety and Health NIOSH, 2011). Chronic pesticide exposure has been associated with autism and attention-deficit/hyperactivity disorder in children (Roberts, et al., 2019).

Here, we present results of an analytical study conducted to assess the label accuracy (based on labels of full spectrum, broad spectrum, and CBD isolate) and purity (based on presence of heavy metals, residual solvents, and pesticides) of 202 commercial CBD products purchased at various web-based retail points of sale from October 2021 to December 2021.

## Materials and methods

### Sample selection

In this study, the selection of CBD products was strategically designed to reflect the typical consumer experience in the United States. Products were purchased from the US open stream of commerce, including brand websites and digital marketplace sources, thereby representing a broad convenience (“retail basket”) sample across a range of price points (see Supplementary Material for further details). These products are neither approved nor regulated by the US Food and Drug Administration. Packaging for each sample was retained, and the list of ingredients, product formulation (i.e., gummy, tincture, topical, or vape), claimed product type (i.e., full spectrum, broad spectrum, isolate, or unspecified), and stated CBD dose were recorded.

### Standard protocol approvals, registrations, and patient consents

This study was exempt from ethics review and informed consent as it did not involve human participants, in accordance with 45 CFR 46.

### Study design

A single sample was tested for each product, and all testing was completed at an International Organization for Standardization (17,025-accredited chemistry laboratory (Ellipse Analytics, Denver, CO, USA). CBD products were tested for label accuracy and product purity. All test methods were validated for each matrix tested (i.e., tincture, lotion, gummy, hemp flower, hemp extract, beverage) using comparable products spiked with appropriate reference standards. The purity of CBD products was benchmarked *versus* standards aggregated from California’s Safe Drinking Water and Toxic Enforcement Act (Proposition 65) safe harbor limits, US Pharmacopeia (USP) safe harbor limits, and Colorado Department of Public Health & Environment (CDPHE) regulations for industrial hemp (USP, 2012; OEHHA, 2021; CDPHE, 2022; USP, 2023). Relevant regulatory limits were gathered for all contaminants of concern detected in the present samples (OEHHA, 2021; CDPHE, 2021; USP 39, 2015).

Label accuracy was assessed by evaluating claims of product type (i.e., “full spectrum,” “broad spectrum,” and “CBD isolate”) on the label (Supplementary Table S1 in Supplement 1). Full spectrum CBD products are derived from hemp extract that do not undergo post-processing to add cannabinoids or completely remove any type of compound; they contain multiple hemp-derived cannabinoids, other naturally occurring compounds, and trace amounts of THC (not >0.3%) (Dahlgren, et al., 2022; Berthold, et al., 2023; OCM, 2024). Our conservative operational definition was presence of CBD, THC, and at least one other phytocannabinoid. Broad spectrum CBD products are also derived from hemp extract and include multiple cannabinoids, but THC has been removed to nondetectable levels during post-processing (Dahlgren, et al., 2022; Berthold, et al., 2023; OCM, 2024); our conservative operational definition was presence of CBD and at least one other phytocannabinoid but no THC. CBD isolate products contain >95% CBD (OCM, 2024); our operational definition for CBD isolate was consistent with this formal definition. Products were considered to exceed, meet, or not meet label claim of CBD potency if they contained >110%, 90%–110%, or <90% of claimed CBD content, respectively (Supplementary Table S1).

Analytes of interest in the evaluation of product purity included 12 naturally occurring phytocannabinoids, four heavy metals, 15 residual solvents, and 232 pesticides. See Supplementary Table S2 for the full list of analytes. As this analysis focused on phytocannabinoid content of products, non-cannabinoid compounds for each sample were not analyzed. Synthetic cannabinoids were analyzed due to reports of significant adverse reactions associated with their use (Cooper, 2016); however, synthetic cannabinoids were not detected in any of the samples. Where applicable to regulatory schema, analytes were examined as micrograms per serving. CBD was measured in milligrams per unit. All other analyses were performed in the most relevant concentration unit (percent weight for cannabinoids, parts per billion for metals and pesticides, and parts per million for residual solvents). Detailed descriptions of extraction and analytical methods are included in Supplementary Material.

### Phytocannabinoid analysis

The cannabinoids analyzed in this study are commonly present in hemp products, have distinct pharmacological profiles, and are detectable via liquid chromatography with diode array detection (LC-DAD). Furthermore, THC levels are critical for regulatory compliance, and several cannabinoids that are not as well studied (e.g., cannabidivarin and tetrahydrocannabivarinic acid) were included in this analysis.

Cannabinoids in CBD samples were extracted into a suitable organic solvent (methanol in most cases). Quality control parameters were met to ensure correct extraction and quantitative accuracy for each analyte.

Extracted cannabinoids were analyzed using LC-DAD. Instrument parameters for the LC-DAD analysis are described in Supplementary Table S3. The measured cannabinoid concentrations were compared with content claims on the original packaging to determine label accuracy.

### Heavy metals analysis

Levels of four heavy metals (cadmium, arsenic, mercury, and lead) that are most commonly associated with poisoning in humans were assessed. Prior to heavy metals analysis, CBD samples were homogenized until a uniform texture was achieved. All standards were prepared using the same diluent as was used for diluting samples. See Supplementary Material for additional details on homogenization and preparation of standards. Levels of heavy metals were assessed using inductively coupled plasma mass spectrometry (ICP-MS; NexION 350, PerkinElmer) (Balali-Mood, et al., 2021; Brooks Applied Labs, 2023). ICP-MS data were processed using Syngistix software (PerkinElmer). Additional details regarding quality control and limits of quantification are provided in Supplementary Material.

### Residual solvents analysis

Samples were analyzed for residual solvents using Thermo Trace 1,300 gas chromatography with flame ionization detection and TriPlus RSH Autosampler and processed with Chromeleon 7.2 software (Thermo Scientific). Supplementary Table S3 shows the parameters used in the residual solvents analysis by gas chromatography.

### Pesticide analysis

Before pesticide analysis, CBD samples were homogenized until a uniform texture was achieved (see Supplementary Material for additional details). Pesticide analysis was conducted using liquid chromatography-tandem mass spectrometry (Supplementary Table S3).

### Statistical analysis

The distribution of heavy metals, residual solvents, and pesticides were examined and benchmarked by both product format and product type. Descriptive statistics were generated, and the concentration of compounds relevant to Proposition 65 were converted from raw concentrations to micrograms per serving for comparison to the standard when CBD serving size was provided on the package (Supplementary Table S4). Results were then compared with the three relevant external benchmarks.

For comparison between the CBD potency claim on the package label with potency measured in the product, we converted the measured concentration of CBD into milligrams per unit. We divided this amount by that listed on the package label to generate a percentage and identified all samples that deviated from 100%, with ±10% deviation considered inaccurate labeling, similar to other label accuracy studies (Bonn-Miller, et al., 2017; Johnson, et al., 2022).

Statistical analysis of the differences in levels of contaminants as a function of format or claimed product type was accomplished by one-way analysis of variance (ANOVA) to determine significant outcomes. Post-hoc comparisons were made between significant groups using Tukey’s honestly significant difference for between-group differences. Analysis of the frequency of pesticide contamination was performed by algorithmic comparison of pesticide detection frequency between product formats. Analysis of CBD label accuracy was performed using ANOVA to determine significant outcomes. Post-hoc comparisons were made between significant groups using Tukey’s honestly significant difference for between-group differences. Comparison of the dispersion of formulation under labeling and over labeling was accomplished using chi-squared analyses for independence. Statistical significance was defined as *p* < 0.05 for all parametric and nonparametric tests.

## Results

### Product labeling accuracy

**FIGURE 1:**
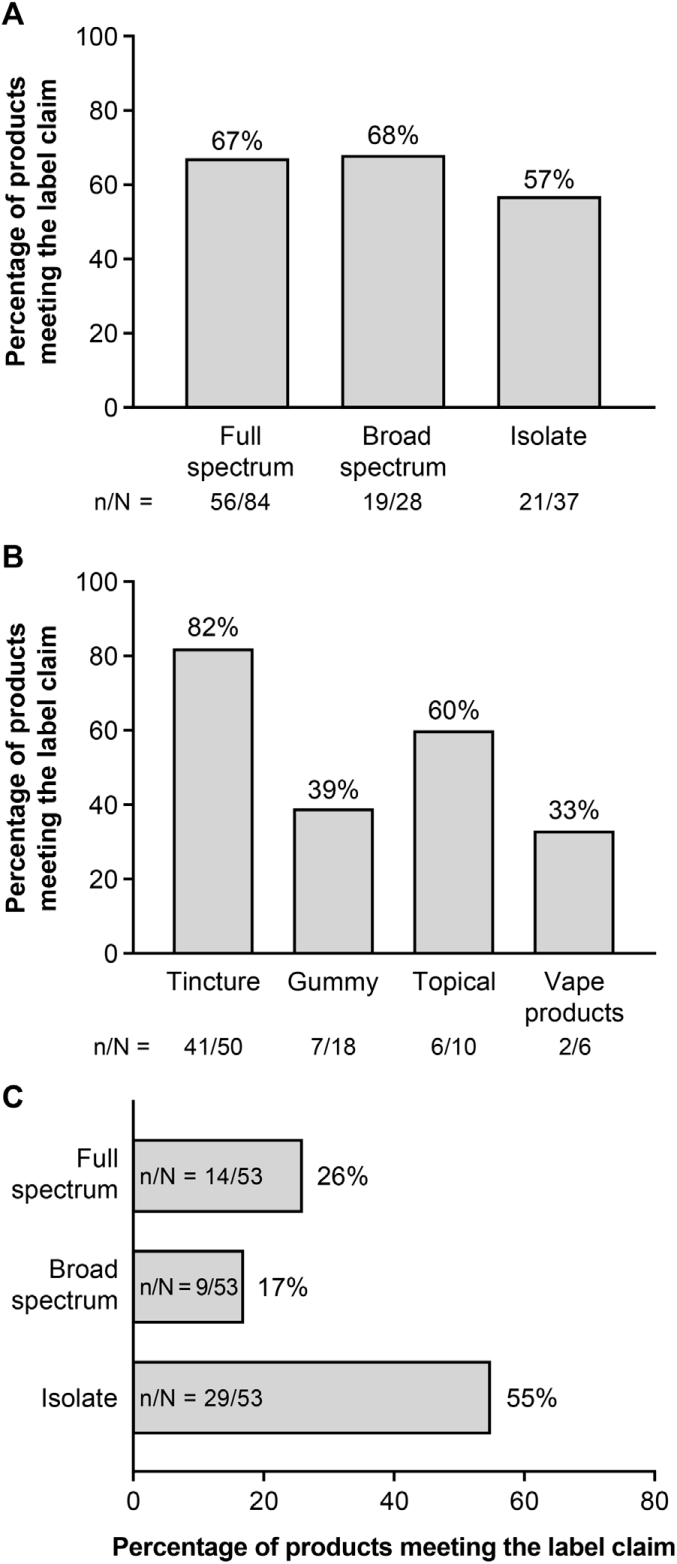
Audit of label claims^a^: **(A)** All products by product type,^b^ **(B)** Full-spectrum products by product format, and **(C)** Unspecified^c^ products by product type. ^a^The audit of the products evaluated the proportion of the analyzed products that conformed with the label claim. Failure to meet the defining criteria could generally be attributed to a lack of phytocannabinoids other than THC; however, five products failed due to a lack of detectable CBD, and three broad-spectrum products had THC, of which two had >0.3% THC. ^b^Full spectrum, broad spectrum, and CBD isolate are based on claims made on the product packaging. ^c^Products did not have a specific claim related to hemp content. CBD, cannabidiol; THC, tetrahydrocannabinol.

Of the 202 CBD products tested, product labels classified 84 as full spectrum, 28 as broad spectrum, and 37 as CBD isolate; 53 products did not have a specific claim (unspecified; Table 1). As shown in Figure 1A, 67% of products labeled full spectrum, 68% of products labeled broad spectrum, and 57% of products labeled CBD isolate met our criteria for each of these product types. There were no significant differences in the proportion of identity-accurate products between the three product formats (*p* = 0.52). Tested products included 100 tinctures, 48 gummies, 34 vape products, and 20 topicals. Among products labeled full spectrum, gummy and vape products were less likely to match their label claim than tinctures or topicals (Figure 1B). Of the 53 unspecified products, 26% met full-spectrum criteria, 17% met broad-spectrum criteria, and 55% were CBD isolates (Figure 1C).

**TABLE 1:** Summary of CBD products tested based on product labeling^a^.

	All products (N = 202)	Tinctures (n = 100)	Gummies (n = 48)	Topicals (n = 20)	Vape products (n = 34)
Full spectrum	84	50	18	10	6
Broad spectrum	28	14	9	4	1
CBD isolate products	37	12	6	2	17
Not specified^b^	53	24	15	4	10
Total	202	100	48	20	34

^a^ Full spectrum, broad spectrum, and CBD, isolate are based on claims made on the product packaging.

^b^ Products did not have a specific claim related to hemp content. CBD, cannabidiol.

In our analysis comparing the CBD potency claim on the package label with measured potency, 46% of the products (93/202) exceeded label claim (contained >110% of claimed CBD content), and 28% (56/202) did not meet label claim (contained <90% of claimed CBD content), for a total of 74% (149/202) of products being under-labeled or over-labeled (Table 2); one product had 565% of the labeled CBD content. There were significant differences in label accuracy as a function of product format (F [3, 198] = 19.804; *p* < 0.001) and claimed product type (F [3, 198] = 4.455; *p* = 0.005). A *post hoc* analysis revealed that gummy products were more likely to be lower than the labeled potency compared with tincture, topical, and vape products (*p* < 0.001). Further, topicals were more likely to have a higher than labeled potency than all other formats (*p* < 0.05). Post-hoc analysis of product type also revealed that isolate products were higher in potency than unspecified products (*p* < 0.05). At least one phytocannabinoid other than THC or CBD was detected in 64% of products. The most commonly occurring “minor” phytocannabinoids were cannabichromene (CBC; 39% [mean, 0.03 mcg (standard deviation [SD], 0.07); range, 0–0.03]), cannabigerol (CBG; 39% [mean, 0.04 mcg (SD, 0.15); range, 0–0.2]), and cannabidivarin (CBDV; 37% [mean, 0.01 mcg (SD, 0.04); range, 0–0.01]). Δ9-THC (mean, 0.04 mcg [SD, 0.22]; range, 0–3.0) and Δ8-THC (mean, 0.03 mcg [SD, 0.26]; range, 0–0) were rarely detected and were found only in low concentrations.

**TABLE 2:** Comparison of CBD potency between package label and product analysis^a^.

Product format	Products exceeding label claim of CBD potency,^b^ n (%)	Products meeting label claim of CBD potency,^c^ n (%)	Products not meeting label claim (low CBD potency),^d^ n (%)
Total	93 (46)	53 (26)	56 (28)
Gummy	4 (2)	17 (8)	27 (13)
Tincture	53 (26)	29 (14)	18 (9)
Topical	18 (9)	1 (<1)	1 (<1)
Vape	18 (9)	6 (3)	10 (5)
Product type^e^			
Total	93 (46)	53 (26)	56 (28)
Broad	15 (7)	6 (3)	7 (3)
Full	39 (19)	22 (11)	23 (11)
Isolate	24 (12)	8 (4)	5 (2)
Not specified^f^	15 (7)	17 (8)	21 (10)

^a^ All percentages are calculated based on a total N = 202.

^b^ >110% claim of CBD, potency.

^c^ 90%–110% claim of CBD, potency.

^d^ <90% claim of CBD, potency.

^e^ Full spectrum, broad spectrum, and CBD, isolate are based on claims made on the product packaging.

^f^ Products did not have a specific claim related to hemp content. CBD, cannabidiol.

### Contamination in CBD products

Heavy metals, residual solvents, and pesticides were found in varying levels in the CBD products tested. A full list of regulatory limits and CBD product violations is provided in Supplementary Table S5. Values for repeatability precision after replicate measurements of various analytes are provided in Supplementary Tables S6−S11.

### Heavy metal contamination

**FIGURE 2:**
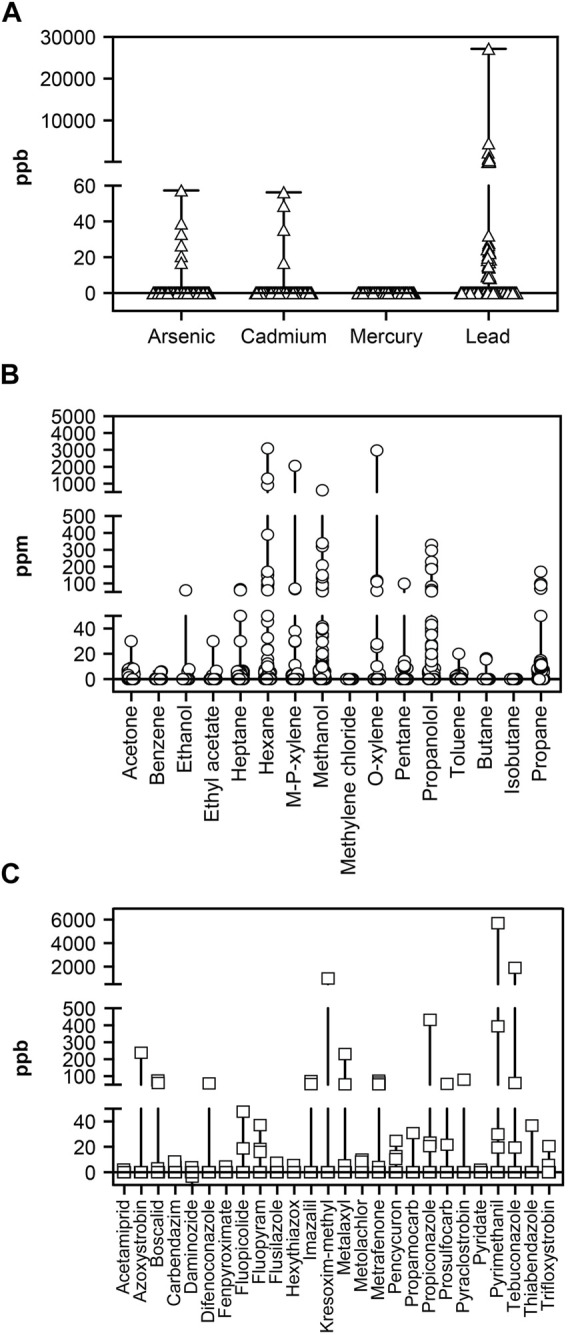
Analysis of **(A)** Heavy metal (ppb), **(B)** Residual solvents (ppm), and **(C)** Pesticide (ppb) contamination in CBD products. Figures show median value and range. Triangles in panel A, circles in panel B, and squares in panel C show individual values. CBD, cannabidiol.

Figure 2A shows results from the analysis of heavy metals, which were detected 52 times across 44 of the 202 products tested (mean, 192.24 ppb [SD, 1943.91]; median not detected [ND], [range, ND–27150]). Lead was the most commonly detected contaminant, with 42 products showing detectable levels (mean, 916.21 ppb [SD, 4222.29]; median, ND [range, ND–27150]), and it was the only heavy metal that violated regulatory (Proposition 65 Safe Harbor and CDPHE) thresholds (5 [3%] products–four tinctures and one gummy formulation). Products with an unspecified product type had significantly more lead than full-spectrum, broad-spectrum, or isolate product types (*p* < 0.001). Arsenic contamination was detected in six samples (mean, 1.06 ppb [SD, 5.97]; median, ND ppb [range, ND–57.40]), and cadmium contamination was detected in four samples (mean, 0.89 ppb [SD, 5.91]; median, ND ppb [range, ND–56.40]). Mercury was ND in any sample.

### Residual solvent contamination

Residual solvents were detected 446 times across 181 of the 202 products; of these, there were 4 (2%) violations of USP safe harbor limits and 19 (9%) violations of CDPHE regulations. The highest concentrations were reported for hexane, m/p-xylene, methanol, & o-xylene, which were also the most commonly detected solvents besides propanol (Figure 2B). There were significant differences in hexane concentration as a function of product format (*F* [3,195], 6.96; *p* < 0.001). Post-hoc analyses demonstrated that vape products showed significantly higher levels of hexane than all other formats (all *p* < 0.05). CBD-isolate products contained more hexane than full-spectrum products (*p* < 0.05). No other significant differences were observed among solvents.

### Pesticide contamination

Figure 2C shows results of pesticide testing. Of 232 pesticides tested, 26 were found 55 times across 30 products. Of the 202 products included in this analysis, there were three (1%) violations of Proposition 65 safe harbor limits and 17 (8%) violations of CDPHE regulations. Due to the low frequency of pesticide contamination, no parametric statistics were performed. Full-spectrum products averaged 0.34 pesticides per product, broad-spectrum products averaged 0.21 pesticides per product, and isolate products averaged 0.16 pesticides per product. Full-spectrum CBD products contained pesticides at 2.12 times the rate of isolate CBD products. The probability that a given product contained a pesticide varied as a function of the amount of CBD in the product (*X* ^2^ (2, 201) = 10.23, *p* < 0.01), with products in the lowest tertile for CBD content being less likely to contain pesticides.

## Discussion

In this study, we examined a large sampling of CBD products of varying formats and tested them for label accuracy and presence of contaminants, including heavy metals, residual solvents, and pesticides. We show that one-third of the products were mislabeled, and heavy metals, residual solvents, and pesticides were present in several products. Although the majority of products had minimal to no contamination, a substantial fraction was contaminated (25% of all products accounted for 93% of total contamination), a concerning finding for consumers who are likely unaware of quality control issues associated with CBD products. Our results are similar to a previously published study that found heavy metal and phthalate contamination in a large sampling of commercially available CBD products in the US, with lead detected in 42%, cadmium in 8%, arsenic in 28%, and mercury in 37% of 121 edible CBD products; phthalate concentrations varied between 13% and 80%, with bis(2-ethylhexyl)phthalate being most prevalent (Gardener, et al., 2022). Contamination can occur at various stages of CBD product preparation. For instance, fungal infections can occur during harvesting and storage under wet/humid conditions, and heavy metals can be introduced prior to harvest via fertilizer uptake, during processing through cross-contamination, and through post-processing adulteration (Dryburgh, et al., 2018).

Nonprescription CBD products remain largely unregulated in the United States, and there are no clear standards for CBD content, labeling accuracy, or lot-to-lot variability (Miller, et al., 2022). In our analysis, 74% of products were found to be either under-labeled or over-labeled (±10% beyond the CBD label claim); one product had 565% of the CBD content mentioned in the label. These findings are consistent with results from other studies conducted in the United States (Wakshlag, et al., 2020; Gardener, et al., 2022; Miller, et al., 2022; Spindle, et al., 2022) and outside of the United States (Pavlovic, et al., 2018; Grafinger, et al., 2020; Liebling, et al., 2020). Our operational definitions of full spectrum and broad spectrum for the purposes of this analysis were more conservative than formal definitions (Fletcher and deLeeuw, 2022; Mutchler, 2022), and since the study focused on cannabinoid content, non-cannabinoid content was not analyzed for full-spectrum or broad-spectrum products. Approximately 40% of the full- and broad-spectrum products did not meet the defining criteria. Failure to meet the criteria could often be attributed to the lack of phytocannabinoids other than THC; however, five products failed because they had no detectable CBD, and three broad-spectrum products had THC, two of which had >0.3% THC.

Discrepancy in product labeling can result in consumption of unknown CBD quantities, which can have deleterious health consequences, especially if CBD products are used *in lieu* of approved medications for serious health concerns. Additionally, understanding metabolic pathways of phytocannabinoids within the human body is becoming increasingly important, particularly when considering potential drug–drug interactions (Amaral Silva, et al., 2020). Phytocannabinoids are extensively metabolized by cytochrome P450 (CYP) enzymes, with CBD as the most potent inhibitor of numerous CYPs, including CYP2C9 and CYP2C19 (Zendulka, et al., 2016). CBD can also be a potent inhibitor of CYP3A and CYP1A2, and CBD–THC interactions can lead to a significant increased risk of AEs (Bansal, et al., 2023; Zamarripa, et al., 2023). Additionally, drug–drug interactions have been reported between CBD and clobazam, as well as between CBD and mammalian target of rapamycin inhibitors (Patsalos, et al., 2020; Wray, et al., 2023).

Another important consideration is the variable effect of CBD depending on concentration and the formulation used. For example, one study found different cannabinoid degradation rates depending on the solvent (Citti, et al., 2016), suggesting the potential for formulation to affect the efficacy of CBD. Although these factors are important, they were not the focus of our current study and additional studies may be needed regarding CBD’s effects based on its concentration and the formulation used. Therefore, for consumer safety, it is important that these products are more stringently regulated and that manufacturers ensure label accuracy by implementing procedures for frequent and thorough independent product testing.

We found that 202 products had 53 regulatory limit violations, with the most common violations against the CDPHE hemp requirements (5 lead, 17 pesticides, and 19 solvents) with four solvent violations also violating USP requirements and an additional eight also violating Proposition 65 requirements (five lead, three solvents). The presence of heavy metals in CBD products has been shown previously (Gardener, et al., 2022) and is concerning from the public health perspective. Although most tested products did not contain detectable lead levels, the products that did contain lead ranged from 8.3 to 27150.0 ppb of lead.

Residual solvents measured in most CBD products in our study are likely remnants from the manufacturing process due to inadequate remediation of solvent(s) (Al Ubeed, et al., 2022). Although hexane, m/p-xylene, and methanol were all present at high mean concentrations in the products tested, the most frequent residual solvent that violated regulation levels was heptane (identified in 51 of 202 products). Pesticides found in multiple products were mostly fungicides, likely because fungus/mold is a common agricultural concern when growing hemp. It is unsurprising, therefore, that “less refined” (i.e., full spectrum) product types contained higher levels of pesticides, as a larger percentage of the original biomass likely remained in the product. Additionally, our pesticide testing panel identified high levels of contamination with pesticides that are not currently required on national or state regulatory testing programs. This suggests the need for a re-evaluation of public policies and guidance for pesticides included in product testing requirements.

In this comprehensive analytical study of a large sample of commercially available CBD products in the US market, which are allowed to be marketed as consumable products under Colorado state law, we show a significant discrepancy in dose label accuracy. Additionally, we found the presence of heavy metals, residual solvents, and pesticides; in some cases, these contaminants were present above levels considered to be safe. Collectively, these findings raise concerns about the quality control of commercially available CBD products, especially considering their increasingly widespread use as treatments for various health conditions, including neurological disorders. This study had some limitations. The samples were not selected at random, and the study did not account for organic or inorganic arsenic. However, the selection of CBD products was strategically designed to reflect the typical consumer experience in the United States and the current market landscape. Furthermore, since only one sample for each product was tested, variability within or between batches could not be evaluated; however, our study results clearly show that uniform compliance with the quality control measures is lacking in the industry. Industry-adopted operational definitions of the terms full spectrum, broad spectrum, and isolate are also required. It is important that the public be aware of these product quality control issues and have informed discussions with their physician when making decisions involving CBD products. Regulatory guidance and enforcement are needed to ensure compliance with product quality standards to protect consumer health and safety.

## Data Availability

The original contributions presented in the study are included in the article/Supplementary Material, further inquiries can be directed to the corresponding author.

## Abbreviations

Δ8-THC, delta-9-tetrahydrocannabinol; Δ9-THC, delta-9-tetrahydrocannabinol; ANOVA, one-way analysis of variance; CBC, cannabichromene; CBD, cannabidiol; CBDa, cannabidiolic acid; CBDV, cannabidivarin; CBG, cannabigerol; CBGa, cannabigerolic acid; CDPHE, Colorado Department of Public Health & Environment; ICP-MS, inductively coupled plasma mass spectrometry; LC-DAD, liquid chromatography with diode array detection; ND, not detected; Proposition 65, California’s Safe Drinking Water and Toxic Enforcement Act; SD, standard deviation; THC, tetrahydrocannabinol; US, United States; USP, US Pharmacopeia.
