# Supplementary material for: Product labeling accuracy and contamination analysis of commercially available cannabidiol product samples
Source: Front Pharmacol. 2024 Mar 18;15:1335441. doi: 10.3389/fphar.2024.1335441 (PMC10982813; doi:10.3389/fphar.2024.1335441)
Supplement: Supplementary file 1 [file DataSheet1.docx]

# Supplemental online content

**Abbreviations for supplement**

ACN, acetonitrile

CBC, cannabichromene

CBD, cannabidiol

CBDa, cannabidiolic acid

CBDV, cannabidivarin

CBDVa, cannabidivarinic acid

CBG, cannabigerol

CBGa, cannabigerolic acid

CBN, cannabinol

CDPHE, Colorado Department of Public Health & Environment

d8-THC, delta-8-tetrahydrocannabinol

d9-THC, delta-9- tetrahydrocannabinol

DAD, diode array detection

DAD, diode array detection

FA, formic acid

GC, gas chromatography

HPLC, high-performance liquid chromatography

LC, liquid chromatography

LC-MS/MS, liquid chromatography with tandem mass spectrometry

ND, not detected

Prop 65, Proposition 65

RSA, residual solvents analysis

THCa, tetrahydrocannabinolic acid

THCV, tetrahydrocannabivarin

USP, United States Pharmacopeia

**Reference for supplement**

ISO. ISO/IEC 17025. 2023; <https://www.iso.org/ISO-IEC-17025-testing-and-calibration-laboratories.html#:~:text=Who%20is%20ISO%2FIEC%2017025,in%20fact%2C%20any%20other%20organization>. Accessed March 01, 2023.

**Supplement 1**

eMETHODS………………………………………………………………………...…pg 3

eTABLE 1. CBD potency and label claims for product type……………………...pg 7

eTABLE 2. Analytes tested…………..………………………………………………pg 8

eTABLE 3. Method parameters for analyte analyses………………………..……pg 10

eTABLE 4. Serving size for CBD products……………………………………..…..pg 12

eTABLE 5. Regulatory limits and identification of CBD product violations…......pg 14

eTABLE 6. Repeatability precision for 12 replicate measurements of hemp
flower analytes……..….………………………………………………………………pg 15

eTABLE 7. Repeatability precision for 12 replicate measurements of hemp
extract analytes…..……………………………………………………………………pg 16

eTABLE 8. Repeatability precision for 12 replicate measurements of CBD
tincture analytes………………………………………………………………….……pg 17

eTABLE 9. Repeatability precision for 12 replicate measurements of CBD
lotion analytes……………………………………………………………….…………pg 18

eTABLE 10. Repeatability precision for 12 replicate measurements of CBD
gummy analytes…………………….…………………………………………………pg 19

eTABLE 11. Repeatability precision for 12 replicate measurements of CBD
beverage analytes..……………………………………………………………………pg 20

This supplementary material has been provided by the authors to give readers additional information about their work.

## eMETHODS

**Sample selection**

Products were selected from among the best sellers identified via market research that was completed in November 2021. Products available in Colorado were purchased and all efforts were made to obtain a sufficient sample size of each product format. Doses and flavors were standardized when possible, but criteria were subject to market availability. Therefore, while not randomized, the sample selection approach was meticulously planned to capture a snapshot of the current market landscape in the United States, catering to the average consumer’s choices and access.

**Sample preparation and analysis**

*Preparation of test portion and extraction*

Samples were prepared prior to analysis according to the Ellipse Lab Sampling for Analyses procedure. The sample preparation and extraction process involved meticulous steps to ensure accuracy and efficiency. Initially, samples were prepped in alignment with Ellipse Lab's established procedures, involving unpacking, thorough homogenization, and storage. Prior to analysis of heavy metals and pesticides, CBD samples were homogenized until a uniform texture was achieved. As described in Gardener et al (Gardener, et al., 2022), in preparation for heavy metal analysis, approximately 0.25 g of homogenized sample was digested with an acid solution of 4 mL nitric acid and 1 mL hydrochloric acid in MARSXpress TFM vessels using a microwave digester (MARS6 One Touch microwave, CEM Corporation). The digest protocol utilized a 20-minute ramp to 200 °C followed by a 20-minute hold at 200 °C and 50-minute cool down to room temperature. Following digestion, the samples were transferred to 50-mL polypropylene tubes and diluted to 40 mL with acid diluent. The diluent consisted of 10% nitric acid, 2% hydrochloric acid, 0.4 % gold, 3% methanol, and internal standards. In preparation for pesticide analysis, approximately 1.0 g of homogenized sample was placed in a 50 mL conical tube. For a 40-ppb calibration standard for pesticides, 15 mL of water was added. During extraction, 5 mL of extraction solution was added to each sample before being vortexed. Tubes were then rotated at 30 rpm for 30 min. One packet of QuEChERS Salts (1.5 g of anhydrous sodium acetate and 6 g of anhydrous magnesium sulfate) were added to each tube as needed and vortexed for 30 seconds. Tubes were then centrifuged at 3,000 rpm for 6 minutes; 8 mL of the supernatant from each tube was transferred to a labeled 15-mL dSPE tube and vortexed again before filtering approximately 2 mL of the supernatant from each tube through a 0.2 μm nylon syringe filter into a labeled autosampler vial. Unless otherwise specified, samples were diluted 10 times and vortexed. Calibrators and initial calibration verifications were created in autosampler vials, vortexed, and stored in a refrigerator.

During extraction, we carefully managed dilution levels, particularly due to the high concentration of target analytes in some products, aiming to limit additional dilutions to a maximum of 10×. Methanol, chosen for its efficiency and cost-effectiveness, served as the primary extraction solvent, negating the need for additives or pH adjustments. For specific products like gummies, the QuEChERS method was utilized, involving a multistep process with water and acetonitrile to effectively separate cannabinoids from other constituents. Each step, from the initial hot water extraction to the final filtration, was precisely executed to ensure the purity and accuracy of our cannabinoid analysis.*Separation*

HPLC is the preferred technology for separating cannabinoids due to the thermal degradation of acidic cannabinoids under gas chromatography conditions. We opted for HPLC over mass spectrometry due to the latter's potential inconsistencies in measurements arising from electrospray ionization. Our method utilizes a finely-tuned C-18 column with a small particle size of 1.9 μm, which ensures a high-resolution and consistent detection of target cannabinoids.

*Measurement*

The detection and measurement of cannabinoids were conducted using a diode array detector, known for its high precision and suitability in identifying all major cannabinoids. We utilized a specific wavelength of 218 nm for measuring target analytes, ensuring a consistent and normalized sensitivity. Quantification of each cannabinoid was against certified standards, and internal standards were incorporated in each sample by the autosampler to ensure consistent instrument performance.

Heavy metal samples were analyzed using kinetic energy distribution mode, which uses energized helium to collide with polyatomic interferences to isolate target elements, improving the accuracy of results. For quality control of heavy metals assessment, the minimum acceptable correlation coefficients for the calibration curve for metals for each run was 0.998, the maximum acceptable percent relative standard deviation for matrix spike duplicates was 15%, and the maximum acceptable variability for the low-level and mid-level calibration verification was 30% and 20%, respectively.^1^ The estimated measurement uncertainty for both analytes was found to be 2.4%, determined by measuring the precision of spiked samples. The limits of quantification were determined to be 8 µg/kg for cadmium, mercury, and lead and 16 µg/kg for arsenic. The method detection limits for this assessment were determined in method blank solutions and were not matrix corrected. The measurement recovery for each matrix was determined using matrix spike and matrix spike duplicates. The values for repeatability precision after 12 replicate measurements were acceptable (within 10% for all values greater than the limit of quantitation) for seven hemp flower analytes (cannabidiolic acid [CBDa], CBGa, CBG, CBD, Δ9-THC, THCa, CBC), 4 hemp extract analytes (CBG, CBD, Δ9-THC, CBC), 1 CBD tincture analyte (CBD), 6 CBD lotion analytes (CBDa, CBG, CBD, Δ9-THC, THCa, CBC), 1 CBD gummy analyte (CBD), and 1 CBD beverage analyte (CBD) (**eTables 5–10 in Supplement**).

Analyses for hemp flower, CBD beverage, and CBD lotion analytes were done as part of the accreditation process and are not the focus of this study; the data are included as supplementary information (**eTables 5–10 in Supplement)** as these data may be useful for researchers in related fields.

**References for Supplement**

GARDENER, H., WALLIN, C. & BOWEN, J. 2022. Heavy metal and phthalate contamination and labeling integrity in a large sample of US commercially available cannabidiol (CBD) products. *Sci. Total Environ,* 851**,** 158110.

## eTABLE 1 CBD potency and label claims for product type.

| **Laboratory findings to confirm label claim** | **Meets label claim of CBD type** | | | **Meets label claim of CBD potency** |
| --- | --- | --- | --- | --- |
|  | **Full spectrum** | **Broad spectrum** | **Isolate** |  |
| Contains CBD | ✓ | ✓ | >95% | N/A |
| Contains other cannabinoids | ✓ | ✓ | Not detectable | N/A |
| Contains THC | ✓ | Not detectable | Not detectable | N/A |
| Measured concentration of CBD is within 90–110% of the concentration listed on the product label | N/A | N/A | N/A | ✓ |

Products were considered to exceed, meet, or not meet label claim of CBD potency if they contained >110%, 90–110%, or <90% of claimed CBD content, respectively.

CBD, cannabidiol; N/A, not applicable; THC, tetrahydrocannabinol.

## eTABLE 2 Analytes tested.

| **Residual solvents** | | |
| --- | --- | --- |
| 2-Propanol  Acetone  Benzene  Butane  Ethanol | Ethyl acetate  Heptane  Hexane  Isobutane  m/p-xylene | Methanol  o-Xylene  Pentane  Propane  Toluene |
| **Heavy metals** | | |
| Arsenic  Cadmium | Mercury  Lead |  |
| **CBD potency** | | |
| CBDVa  CBDV  CBDa  CBGa | CBG  CBD  THCV  CBN | d9-THC  d8-THC  THCa  CBC |
| **Pesticides** | | |
| 3-Hydroxycarbofuran  Acephate  Acetamiprid  Aldicarb  Aldicarb sulfone  Aldicarb sulfoxide  Ametryn  Atrazine  Avermectin B1A  Azaconazole  Azinphos-ethyl  Azoxystrobin  Benalaxyl  Bendiocarb  Benthiavalicarb-isopropyl  Bioresmethrin  Bitertanol  Boscalid  Bromacil  Bromoxynil  Bupirimate  Buprofezin  Butralin  Cadusafos  Carbaryl  Carbendazim  Carbetamide  Carbofuran  Carboxin  Chlorantraniliprole  Chlorfenvinphos  Chlorotoluron  Chloroxuron  Chlorpyrifos  Clodinafop-propargyl ester  Clofentezine  Clopyralid  Cloquintocet-1-methylhexyl ester  Clothianidin  Crimidine  Cyanazine  Cyazofamid  Cycloate  Cycloxydim  Cyprodinil  Cyromazine  Daminozide  DEET  Deltamethrin  Demeton-S-methyl  Demeton-S-methyl sulfone  Desmetryne  Diafenthiuron  Diallate (total)  Diazinon  Dichlorvos  Diethofencarb  Difenoconazole  Diflubenzuron  Dimethoate  Dimethomorph  Diniconazole  Dinoseb  Dinotefuran  Diphenamid  Diphenylamine  Disulfoton  Disulfoton sulfone  Disulfoton-sulfoxide  Diuron  Dyrene  Emamectin B1A  Epoxiconazole  EPTC  Ethiofencarb  Ethiofencarb sulfoxide  Ethion  Ethiprole | Ethirimol  Ethoprophos  Ethoxyquin  Etoxazole  Fenamidone  Fenamiphos  Fenamiphos sulfone  Fenamiphos sulfoxide  Fenarimol  Fenazaquin  Fenbuconazole  Fenhexamid  Fenoxaprop-P  Fenpropimorph  Fenpyroximate  Fenthion  Fenthion oxon  Fenthion Sulfone  Fipronil  Flamprop-M-isopropyl  Flonicamid  Fluazifop  Fluazifop-P-butyl  Fludioxonil  Flufenacet  Fluopicolide  Fluopyram  Fluoxastrobin  Fluquinconazole  Fluridone  Flurprimidol  Flusilazole  Fluthiacet-methyl  Flutolanil  Flutriafol  Fonofos  Forchlorfenuron  Fosthiazate  Fuberidazole  Furalaxyl  Haloxyfop (free acid)  Hexaconazole  Hexazinone  Hexythiazox  Icaridin  Imazalil  Imidacloprid  Indoxacarb  Iprovalicarb  Isofenphos  Isoprocarb  Isopropalin  Isoproturon  Kresoxim-methyl  Lenacil  Linuron  Malaoxon  Malathion  Mandipropamid  Mecarbam  Mepanipyrim  Mepronil  Metalaxyl  Metamitron  Metazachlor  Methabenzthiazuron  Methamidophos  Methidathion  Methiocarb  Methomyl  Methoxyfenozide  Metobromuron  Metolachlor  Metrafenone  Metribuzin  Molinate  Monolinuron | Myclobutanil  Napropamide  Neburon  Nitenpyram  Norflurazon  Nuarimol  Ofurace  Omethoate  Oxadixyl  Oxydemeton-methyl  Penconazole  Pencycuron  Pethoxamid  Phorate sulfoxide  Picoxystrobin  Pirimicarb  Pirimicarb-desmethyl  Pirimiphos-ethyl  Pirimphos-methyl  Prochloraz  Profoxydim-lithium  Promecarb  Prometryn  Pronamide  Propamocarb  Propaquizafop  Propargite  Propiconazole  Propoxur  Propoxycarbazone  Proquinazid  Prosulfocarb  Pymetrozine  Pyraclostrobin  Pyridaben  Pyridaphenthion  Pyridate  Pyrimethanil  Pyriproxyfen  Quinclorac  Quinmerac  Quinoxyfen  Quizalofop  Resmethrin  Rotenone  Sethoxydim  Simazine  Spinosyn A  Spinosyn D  Spiromesifen  Tebuconazole  Tebufenozide  Tebufenpyrad  Tepraloxydim  Terbufos sulfone  Terbufos sulfoxide  Terbuthylazine  Terbutryn  Tetrachlorvinphos  Tetraconazole  Thiabendazole  Thiacloprid  Thiadicarb  Thiamethoxam  Thiodicarb  Thiofanox-sulfone  Tralkoxydim  Triadimefon  Triadimenol  Tribenuron-methyl  Trichlorfon  Tricyclazole  Trifloxystrobin  Triflumizole  Triflumuron  Triticonazole  Zoxamide |

CBC, cannabichromene; CBD, cannabidiol; CBDa, cannabidiolic acid; CBDV, cannabidivarin; CBDVa, cannabidivarinic acid; CBG, cannabigerol; CBGa, cannabigerolic acid; CBN, cannabinol; d8-THC, delta-8-tetrahydrocannabinol, d9-THC, delta-9-tetrahydrocannabinol; THCa, tetrahydrocannabinolic acid; THCV, tetrahydrocannabivarin

## eTABLE 3 Method parameters for analyte analyses.

| **Cannabinoid analysis by LC-DAD** | |
| --- | --- |
| Column | Agilent Poroshell 120 EC-C18 (100 mm × 2.1 mm × 1.9 μm) |
| Column temperature | 40 °C |
| Injection volume | 1 μL - with injector programming |
| Flow rate | 0.8 mL/minute |
| Run time | 8 minutes |
| Organic mobile phase (C line) | LC-MS ACN with 0.1% FA |
| Aqueous mobile phase (D Line) | 10 mM NH_4_HCO_2_ in H_2_O, pH 3.6 with FA |
| Aqueous wash (A line) | H_2_O |
| Organic wash (B line) | LC-MS ACN |
| **RSA by GC** | |
| Helium gas flow rate | 2.0 mL/minute |
| Inlet temperature | 280 °C |
| Inlet split | 5 |
| Liner type | Splitless liner with glass wool |
| **Pesticide analysis by LC-MS/MS** | |
| Column | Restek Raptor ARC-18 2.7 μm 150 x 3.0 mm |
| Column temperature | 40 °C |
| Injection volume | 10 μL |
| Flow rate | 0.6 mL/minute |
| LC stop time | 23 minutes |
| Aqueous mobile phase (A line) | 5 mM ammonium formate with 0.1% FA |
| Organic mobile phase (B line) | Methanol with 0.1% FA |

ACN, acetonitrile; FA, formic acid; DAD, diode array detection; GC, gas chromatography; LC, liquid chromatography; LC-MS/MS, liquid chromatography with tandem mass spectrometry; RSA, residual solvents analysis

## eTABLE 4 Serving size for CBD products.

| Product format | Serving listed | Count of serving listed |
| --- | --- | --- |
| Gummy |  | 48 |
|  | Yes | 48 |
| Tincture |  | 100 |
|  | No | 2 |
|  | Yes | 98 |
| Topical |  | 20 |
|  | No | 20 |
| Vape |  | 34 |
|  | No | 18 |
|  | Yes | 16 |
| Total |  | 202 |

CBD, cannabidiol

## eTABLE 5 Regulatory limits and identification of CBD product violations.

| **Contaminant type** | **Contaminant** | **Prop 65** | | **USP** | | **CDPHE** | |
| --- | --- | --- | --- | --- | --- | --- | --- |
|  |  | **Limit (ug/day)** | **Violations, n (%)** | **Limit (mg/day)** | **Violations, n (%)** | **Limit (ppm)** | **Violations, n (%)** |
| Heavy metals | Arsenic | 10.0 | 0 | 0.015 | 0 | 1.5 | 0 |
|  | Cadmium | 4.1 | 0 | 0.005 | 0 | 0.5 | 0 |
|  | Mercury | 0.3 | 0 | 0.03 | 0 | 1.5 | 0 |
|  | Lead | 0.5 | 5 (3) | 0.005 | 0 | 0.5 | 5 (3) |
| Residual solvents | Acetone |  | 0 | 50 | 0 | 1000 | 0 |
|  | Benzene | 6.4 | 0 | 2 | 2 (1) | 2 | 2 (1) |
|  | Butane |  | 0 |  | 0 | 1000 | 0 |
|  | Ethanol |  | 0 | 50 | 0 | 1000 | 0 |
|  | Ethyl acetate |  | 0 | 50 | 0 | 1000 | 0 |
|  | Heptane |  | 0 | 50 | 0 | 1000 | 0 |
|  | Hexane | 28,000 | 0 | 2.9 | 0 | 60 | 15 (8) |
|  | Isobutane |  | 0 |  | 0 | 1000 | 0 |
|  | mp-xylene |  | 0 | 21.7 | 0 | 430 | 1 (1) |
|  | Methanol | 23,000 | 0 | 30 | 0 | 600 | 0 |
|  | o-xylene |  | 0 | 21.7 | 0 | 430 | 1 (1) |
|  | Pentane |  | 0 | 50 | 0 | 1000 | 0 |
|  | Propane |  | 0 | 50 | 0 | 1000 | 0 |
|  | Propanol |  | 0 | 50 | 0 |  | 0 |
|  | Toluene | 7000 | 0 | 8.9 | 0 | 180 | 0 |
| Pesticides | Acetamiprid |  | 0 |  | 0 | 0.05 | 0 |
|  | Azoxystrobin |  | 0 |  | 0 | 0.01 | 1 (1) |
|  | Boscalid |  | 0 |  | 0 | 0.01 | 3 (2) |
|  | Carbendazim |  | 0 |  | 0 |  | 0 |
|  | Daminozide | 40.0 | 0 |  | 0 | ND | 0 |
|  | Difenoconazole |  | 0 |  | 0 |  | 0 |
|  | Fenpyroximate |  | 0 |  | 0 | 0.02 | 0 |
|  | Fluopicolide |  | 0 |  | 0 |  | 0 |
|  | Fluopyram |  | 0 |  | 0 | 0.01 | 3 (2) |
|  | Flusilazole |  | 0 |  | 0 |  | 0 |
|  | Hexythiazox |  | 0 |  | 0 | 0.01 | 0 |
|  | Imazalil | 11.0 | 0 |  | 0 | 0.01 | 2 (1) |
|  | Kresoxim-methyl | 0^a^ | 1 (1) |  | 0 | 0.15 | 0 |
|  | Metalaxyl |  | 0 |  | 0 | 0.01 | 2 (1) |
|  | Metolachlor |  | 0 |  | 0 |  | 0 |
|  | Metrafenone |  | 0 |  | 0 |  | 0 |
|  | Pencycuron |  | 0 |  | 0 |  | 0 |
|  | Propamocarb |  | 0 |  | 0 |  | 0 |
|  | Propiconazole |  | 0 |  | 0 | ND | 1 (1) |
|  | Prosulfocarb |  | 0 |  | 0 |  | 0 |
|  | Pyraclostrobin |  | 0 |  | 0 | 0.01 | 1 (1) |
|  | Pyridate |  | 0 |  | 0 |  | 0 |
|  | Pyrimethanil |  | 0 |  | 0 |  | 0 |
|  | Tebuconazole |  | 0 |  | 0 | 0.01 | 2 (1) |
|  | Thiabendazole |  | 1 (1) |  | 1 (1) | 0.02 | 1 (1) |
|  | Trifloxystrobin |  | 1 (1) |  | 1 (1) | 0.01 | 1 (1) |

^a^This product is listed under Prop 65 but has no safe harbor limit.

CBD, cannabidiol; CDPHE, Colorado Department of Public Health & Environment; ND, not detected; Prop 65, Proposition 65; USP, United States Pharmacopeia

## eTable 6 Repeatability precision for 12 replicate measurements of hemp flower analytes.^a^

| Analyte measured | Average (mg/g) | RSD_r_ (%) | Pass/fail |
| --- | --- | --- | --- |
| CBDVa | Not detected | - | - |
| CBDV | Not detected | - | - |
| CBDa | 54.50 | 5.06 | Pass |
| CBGa | 1.09^b^ | 5.62 | Pass |
| CBG | 0.45^b^ | 6.67 | Pass |
| CBD | 10.50 | 4.61 | Pass |
| THCV | Not detected | - | - |
| CBN | Not detected | - | - |
| Δ9-THC | 0.92^b^ | 5.20 | Pass |
| Δ8-THC | Not detected | - | - |
| THCa | 1.11 | 6.46 | Pass |
| CBC | 1.26 | 6.47 | Pass |

^a^Acceptable values of repeatability precision were 10% for all values greater than the limit of quantitation.

^b^Measured value was less than the limit of quantitation.

Δ8-THC, delta-8-tetrahydrocannabinol; Δ9-THC, delta-9- tetrahydrocannabinol; CBC, cannabichromene; CBD, cannabidiol; CBDa, cannabidiolic acid; CBDV, cannabidivarin; CBDVa, cannabidivarinic acid; CBG, cannabigerol; CBGa, cannabigerolic acid; CBN, cannabinol; RSDr, relative standard deviation for repeatability; THCa, tetrahydrocannabinolic acid; THCV, tetrahydrocannabivarin

## eTABLE 7 Repeatability precision for 12 replicate measurements of hemp extract analytes.^a^

| Analyte measured | Average (mg/g) | RSD_r_ (%) | Pass/fail |
| --- | --- | --- | --- |
| CBDVa | Not detected | - | - |
| CBDV | Not detected | - | - |
| CBDa | Not detected | - | - |
| CBGa | Not detected | - | - |
| CBG | 11.01 | 6.53 | Pass |
| CBD | 253.4 | 6.00 | Pass |
| THCV | Not detected | - | - |
| CBN | Not detected | - | - |
| Δ9-THC | 19.49 | 7.00 | Pass |
| Δ8-THC | Not detected | - | - |
| THCa | Not detected | - | - |
| CBC | 17.55 | 8.01 | Pass |

^a^Acceptable values of repeatability precision were 10% for all values greater than the limit of quantitation.

Δ8-THC, delta-8-tetrahydrocannabinol; Δ9-THC, delta-9- tetrahydrocannabinol; CBC, cannabichromene; CBD, cannabidiol; CBDa, cannabidiolic acid; CBDV, cannabidivarin; CBDVa, cannabidivarinic acid; CBG, cannabigerol; CBGa, cannabigerolic acid; CBN, cannabinol; RSDr, relative standard deviation for repeatability; THCa, tetrahydrocannabinolic acid; THCV, tetrahydrocannabivarin

## eTABLE 8 Repeatability precision for 12 replicate measurements of CBD tincture analytes.^a^

| Analyte measured | Average (mg/g) | RSD_r_ (%) | Pass/fail |
| --- | --- | --- | --- |
| CBDVa | Not detected | - | - |
| CBDV | Not detected | - | - |
| CBDa | Not detected | - | - |
| CBGa | Not detected | - | - |
| CBG | Not detected | - | - |
| CBD | 11.08 | 4.71 | Pass |
| THCV | Not detected | - | - |
| CBN | Not detected | - | - |
| Δ9-THC | Not detected | - | - |
| Δ8-THC | Not detected | - | - |
| THCa | Not detected | - | - |
| CBC | Not detected | - | - |

^a^Acceptable values of repeatability precision were 10% for all values greater than the limit of quantitation.

Δ8-THC, delta-8-tetrahydrocannabinol; Δ9-THC, delta-9- tetrahydrocannabinol; CBC, cannabichromene; CBD, cannabidiol; CBDa, cannabidiolic acid; CBDV, cannabidivarin; CBDVa, cannabidivarinic acid; CBG, cannabigerol; CBGa, cannabigerolic acid; CBN, cannabinol; RSDr, relative standard deviation for repeatability; THCa, tetrahydrocannabinolic acid; THCV, tetrahydrocannabivarin

## eTABLE 9 Repeatability precision for 12 replicate measurements of CBD lotion analytes.^a^

| Analyte measured | Average (mg/g) | RSD_r_ (%) | Pass/fail |
| --- | --- | --- | --- |
| CBDVa | Not detected | - | - |
| CBDV | Not detected | - | - |
| CBDa | 0.32 | 8.00 | Pass |
| CBGa | Not detected | - | - |
| CBG | 0.11^b^ | 10.90 | Pass |
| CBD | 9.06 | 4.93 | Pass |
| THCV | Not detected | - | - |
| CBN | Not detected | - | - |
| Δ9-THC | 0.23^b^ | 4.55 | Pass |
| Δ8-THC | Not detected | - | - |
| THCa | 0.07^b^ | 16.02 | Pass |
| CBC | 0.30 | 6.37 | Pass |

^a^Acceptable values of repeatability precision were 10% for all values greater than the limit of quantitation.

^b^Measured value was less than the limit of quantitation.

Δ8-THC, delta-8-tetrahydrocannabinol; Δ9-THC, delta-9- tetrahydrocannabinol; CBC, cannabichromene; CBD, cannabidiol; CBDa, cannabidiolic acid; CBDV, cannabidivarin; CBDVa, cannabidivarinic acid; CBG, cannabigerol; CBGa, cannabigerolic acid; CBN, cannabinol; RSDr, relative standard deviation for repeatability; THCa, tetrahydrocannabinolic acid; THCV, tetrahydrocannabivarin

## eTABLE 10 Repeatability precision for 12 replicate measurements of CBD gummy analytes.^a^

| Analyte measured | Average (mg/g) | RSD_r_ (%) | Pass/fail |
| --- | --- | --- | --- |
| CBDVa | Not detected | - | - |
| CBDV | Not detected | - | - |
| CBDa | Not detected | - | - |
| CBGa | Not detected | - | - |
| CBG | Not detected | - | - |
| CBD | 0.86 | 5.04 | Pass |
| THCV | Not detected | - | - |
| CBN | Not detected | - | - |
| Δ9-THC | Not detected | - | - |
| Δ8-THC | Not detected | - | - |
| THCa | Not detected | - | - |
| CBC | Not detected | - | - |

^a^Acceptable values of repeatability precision were 10% for all values greater than the limit of quantitation.

Δ8-THC, delta-8-tetrahydrocannabinol; Δ9-THC, delta-9- tetrahydrocannabinol; CBC, cannabichromene; CBD, cannabidiol; CBDa, cannabidiolic acid; CBDV, cannabidivarin; CBDVa, cannabidivarinic acid; CBG, cannabigerol; CBGa, cannabigerolic acid; CBN, cannabinol; RSDr, relative standard deviation for repeatability; THCa, tetrahydrocannabinolic acid; THCV, tetrahydrocannabivarin

## eTABLE 11 Repeatability precision for 12 replicate measurements of CBD beverage analytes.^a^

| Analyte measured | Average (mg/g) | RSD_r_ (%) | Pass/fail |
| --- | --- | --- | --- |
| CBDVa | Not detected | - | - |
| CBDV | Not detected | - | - |
| CBDa | Not detected | - | - |
| CBGa | Not detected | - | - |
| CBG | Not detected | - | - |
| CBD | 0.089 | 4.32 | Pass |
| THCV | Not detected | - | - |
| CBN | Not detected | - | - |
| Δ9-THC | Not detected | - | - |
| Δ8-THC | Not detected | - | - |
| THCa | Not detected | - | - |
| CBC | Not detected | - | - |

^a^Acceptable values of repeatability precision were 10% for all values greater than the limit of quantitation.

CBC, cannabichromene; CBD, cannabidiol; CBDa, cannabidiolic acid; CBDV, cannabidivarin; CBDVa, cannabidivarinic acid; CBG, cannabigerol; CBGa, cannabigerolic acid; CBN, cannabinol; RSDr, relative standard deviation for repeatability; THCa, tetrahydrocannabinolic acid; THCV, tetrahydrocannabivarin
